# Action of Betulinic Acid in the Inhibition of Efflux Pump NorA in *Staphylococcus aureus* Strains: In Vitro and In Silico Approaches

**DOI:** 10.1002/cbdv.202502869

**Published:** 2025-12-17

**Authors:** Camila Aparecida Pereira da Silva, Nara juliana Santos Araujo, Cícera Datiane Morais Oliveira‐Tintino, José Maria Barbosa Filho, Gabriel Gonçalves Alencar, José Bezerra de Araújo‐Neto, Josefa Sayonara dos Santos, Juliete Bezerra Soares, Carolina Bandeira Domiciano, Henrique Douglas Melo Coutinho, Jacqueline Cosmo Andrade‐Pinheiro

**Affiliations:** ^1^ Graduate Program in Health Sciences Federal University of Cariri Barbalha Brazil; ^2^ Laboratory of Applied Microbiology–LAMAP Federal University of Cariri Barbalha Brazil; ^3^ Multicenter Postgraduate Program in Biochemistry and Molecular Biology Federal University of Cariri Barbalha Brazil; ^4^ Laboratory of Microbiology and Molecular Biology Regional University of Cariri – LMBM Crato Brazil; ^5^ Federal University of Paraiba – UFPB João Pessoa Brazil; ^6^ Graduate Program in Biological Sciences, Biosciences Center Federal University of Pernambuco Recife Brazil

**Keywords:** betulinic acid, efflux pump, pentacyclic triterpene, resistance, *Staphylococcus aureus*

## Abstract

Antibiotic resistance poses a serious challenge to public health, particularly in the case of *Staphylococcus aureus*, a Gram‐positive bacterium that employs multiple resistance mechanisms, including efflux pumps such as NorA, which extrude antimicrobial compounds from the cell and reduce antibiotic efficacy. Therefore, the search for substances capable of inhibiting these mechanisms represents a promising strategy to combat bacterial resistance. Betulinic acid (BA), a pentacyclic triterpene of the lupane type, commonly found in different parts of plants, has demonstrated various pharmacological activities, including antibacterial effects. This study investigated, through in vitro and in silico analyses, the inhibitory action of BA on the NorA efflux pump in *S. aureus* strains SA‐1199 and SA‐1199B. The minimum inhibitory concentrations (MICs) were determined using the broth microdilution method. Subsequently, their effects on efflux pump‐mediated antibiotic resistance were evaluated by reducing the MIC of the antibiotic and ethidium bromide (EtBr), while fluorimetry and permeability potential tests were carried out using the SYTOX Green fluorescence method. Although BA did not show intrinsic antibacterial activity, showing MIC ≥ 1024 µg/mL, it was able to decrease the MIC of norfloxacin and EtBr, as well as influence membrane permeability and increase fluorescence emission. The results, therefore, indicate that BA has considerable potential as an efflux pump inhibitor and could help in the treatment of resistant bacterial infections.

## Graphical abstract

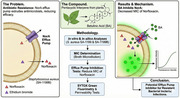

## 1 Introduction

Bacterial infections caused by resistant microorganisms have a significant impact on global public health [1]. This problem is aggravated by the declining efficacy of antibiotics, the result of the constant development of molecular mechanisms that undermine antibiotic activity [2]. Moreover, studies indicate that the use of antibiotics can alter the composition of the microbiota, favoring the growth of resistant pathogens [3]. Among the bacteria associated with resistance and persistence of infections, *Staphylococcus aureus* stands out [4]. This pathogen is responsible for various community and hospital infections, with potentially fatal complications, such as bacteremia, endocarditis, meningitis, and liver abscess [5]. *S. aureus* has significant pathogenicity potential due to multiple virulence factors expressed concurrently [6].

Recognizing the significant impact of *S. aureus* on serious infections, in 2017, the World Health Organization included this pathogen on its list of priorities for the development of new antibiotics, classifying it as a high priority [7]. One of the main resistance mechanisms of *S. aureus* is the expression of efflux pumps. These membrane proteins perform various functions in bacterial pathogenesis, such as regulating chemical reactions within the cell, resisting antibiotics by expelling structurally distinct compounds, and decreasing their intracellular concentration, preventing them from reaching their biological targets [8, 9].

The NorA efflux pump is one of the most investigated efflux systems in *S. aureus* and is associated with the microorganism's multidrug resistance, being overexpressed in 43% of the strains [10, 11], and has a wide range of substrates, such as fluoroquinolones, dyes, antiseptics, and quaternary ammonium compounds. Using the driving force of protons, this pump expels these compounds from the cell [12].

Research into compounds of natural origin to fight infections is increasingly being stimulated due to the decreased susceptibility of microorganisms to the therapeutics used in currently available treatments [13]. In addition, plant‐based products have a lower incidence of side effects compared to synthetic medicines [14]. Triterpenes are a broad class of natural compounds with around 30,000 identified structures [15]. These compounds represent an important group of organic lipid compounds that play significant structural and functional roles in plants [16]. Triterpenoids are commonly found in nature in tetra or pentacyclic forms, although acyclic, monocyclic, bicyclic, tricyclic, and hexacyclic variations also exist [15].

Betulinic acid (BA), or 3‐hydroxy‐lup‐20(29)‐en‐28‐oic acid, is a pentacyclic triterpene of the lupane type, present in stem bark, leaves, and fruit peel [17]. Although it is found in greater concentration in the bark of the Betula tree, other plant species such as *Silene suculenta Forssk*., *Calothamnus quadrifidus*, and *Sarcomphalus joazeiro*, also contain BA in their composition [13, 18, 19]. BA and its derivatives have demonstrated a series of pharmacological activities, such as analgesic, anti‐HIV [20], antineoplastic, anti‐inflammatory, antifungal [21], and antibacterial potential [18].

In view of the above, this study aimed to investigate, in vitro and in silico, the inhibitory action of BA against efflux pumps in strains of *S. aureus*, SA‐1199 and SA‐1199B, which express the efflux protein NorA.

## 2 Materials and Methods

### 2.1 Drugs, Substances, and Strains

BA, (C_30_H_48_O_3_), was purchased from Sigma Aldrich Co. Ltd. (Darmstadt, Germany) with purity >90%. The substances used, norfloxacin, carbonylcyanide 3‐chlorophenylhydrazone (CCCP), ethidium bromide (EtBr), and the dye SYTOX Green were obtained from Sigma Aldrich Co. Ltd. (Darmstadt, Germany). In the assays were used the strains of *S. aureus* SA‐1199 (low expression), SA‐1199B, which overexpress the NorA efflux protein. These strains were maintained on blood agar (Laboratories Difco Ltda., São Paulo‐SP, Brazil) and, prior to the experiments, all bacterial strains were cultured for 24 h at 37°C on Brain Heart Infusion‐Agar (BHI‐Agar, Acumedia Manufacturers Inc., India)

### 2.2 Determination of the Minimum Inhibitory Concentration

The minimum inhibitory concentration (MIC) was determined using the broth microdilution method with modifications [22, 23]. The strains were seeded on HIA 24 h prior to the experiments. After this period, the bacterial inoculum was suspended in saline solution, corresponding to 0.5 on the McFarland scale (1.5 × 10⁸). The Eppendorf tubes were filled with 900 µL of BHI and 100 µL of the inoculum, and the microplates were loaded with 100 µL of the final solution.

The microdilution was performed with 100 µL of the test compound in serial dilutions up to the penultimate well of the plate (1:1), with the last well serving as the growth control. Readings were taken after 24 h of incubation, followed by the addition of 20 µL of resazurin. A color change from blue to pink was interpreted as indicative of bacterial growth [24]. All tests were performed in triplicate.

### 2.3 Evaluation of Efflux Pump Inhibition by Reducing the MIC and Fluorescence of Antibiotics and EtBr

24 h before the test, the *S. aureus* 1199B strain was seeded on Mueller‐Hinton Agar culture medium and then incubated in a bacteriological oven at 37°C. The bacterial inoculum was prepared in phosphate‐buffered saline (PBS) buffer according to the McFarland scale index of 0.5. Solutions were then prepared containing the bacterial inoculum and BA at 50 µg/mL or CCCP at 50 µg/mL, used as a positive control. The negative control consisted only of PBS and bacterial inoculum. These solutions were incubated for 1h30min. Afterwards, 100 µg/mL EtBr was added to all the solutions, and they were kept again in a bacteriological oven. The solutions were then centrifuged at 10 000 rpm for 2 min and washed with PBS, discarding the supernatant, until all the EtBr and remaining substances were removed. The pellet was dissolved in PBS and distributed in black microplates. Reading was carried out with a fluorescence microplate reader (Cytation 1; BioTek, Winooski, VT, USA) and Gen5 3.22 Software, using excitation at 530 nm and emission wavelength 590 nm. The test was performed in triplicate [23, 25].

### 2.4 Evaluation of Bacterial Membrane Permeability Using the SYTOX Green Fluorescence Method

SYTOX Green dye was used for this test. The inoculum of *S. aureus* 1199B was prepared according to the McFarland scale standard of 0.5. The inoculum was distributed in a 96‐well black plate. BA was then added to obtain final concentrations of 200, 100, and 50 µg/mL. Polymyxin B was used as a positive control at the final concentrations of 100, 50, and 25 µg/mL. In the negative control, only PBS was added to the inoculum. The plates were incubated for 1 h. Afterwards, 100 µL of SYTOX Green was added to each well until the final concentration of 1 µM was obtained, then the plates were incubated for 30 min. Reading was carried out using the Cytation 1 fluorescence reader, BioTek (Winooski, VT, USA), and Gen5 3.11 software. 485 nm excitation and 528 nm emission filters were used. The tests were carried out in triplicate [23, 26].

### 2.5 Molecular Docking

With the NorA sequence (P0A0J7) retrieved from the UniProt database (https://www.uniprot.org/), the protein model was generated in SWISS‐MODEL [27], with PDB: 7lo8 as the template (99.74% sequence identity). The NorA model was then evaluated using the SWISS‐MODEL tools and the SAVES v.6.1 server (https://saves.mbi.ucla.edu/), and was deemed valid for docking simulations (Supporting Information). The structures of BA, CCCP, norfloxacin, and EtBr were drawn in MarvinSketch 23.14 (Chemaxon) and optimized in Open Babel 2.4.1 (force field MMFF94). No AutoDock Tools (https://ccsb.scripps.edu/mgltools/), protein and ligands were prepared for docking by adding hydrogens (mixing non‐polar), Gasteiger charges, and flexibility. The NorA components on which flexibility was applied were the 11 residues highlighted in the study by Brawley et al. AutoDock Vina 1.2.5 [28] was used for the docking experiments. The grid box had dimensions (x, y, and z) of 30 Å and was inserted in the center of NorA (x: 137.57, y: 136.065, z: 157.989). The exhaustiveness was set at 32, and the other parameters were set as defaults.

### 2.6 Statistical Analyses

Graphpad Prism software, version analysis, applying one‐way analysis of variance. The results were expressed as arithmetic mean, and standard deviation followed by the Dunnett post hoc, and the results were considered significant when p < 0.05, and not significant when *p* > 0.05.

## 3 Results

### 3.1 Antibacterial Activity of BA

BA did not exhibit clinically relevant antibacterial activity against *S. aureus* strains ATCC 25923, 1199, and 1199B. MIC values are shown in Table 1.

**TABLE 1:** Minimum inhibitory concentration (MIC) of betulinic acid (BA) against *Staphylococcus aureus* strains.

*Staphylococcus aureus* strain	MIC (µg/mL)
ATCC 25923	≥ 1024
1199	≥ 1024
1199B	≥ 1024

The data obtained indicate that BA has no intrinsic action as an antibacterial agent against *S. aureus* strains expressing the NorA efflux pump.

### 3.2 Evaluation of Efflux Pump Inhibition by Reducing the MIC of Antibiotic and EtBr

**FIGURE 1:**
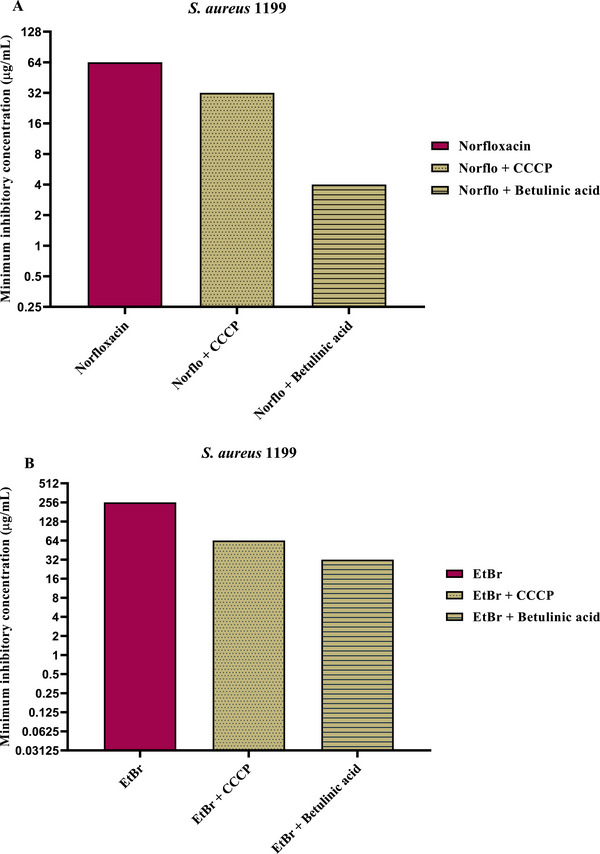
Evaluation of NorA efflux pump inhibition by Betulinic acid against the *S. aureus* 1199 strain. Substance associated with norfloxacin (A) and EtBr (B) in minimum inhibitory concentration (MIC) reduction. Norflo = Norfloxacin, CCCP = Carbonyl cyanide m‐chlorophenyl hydrazone, EtBr = Ethidium bromide.

**FIGURE 2:**
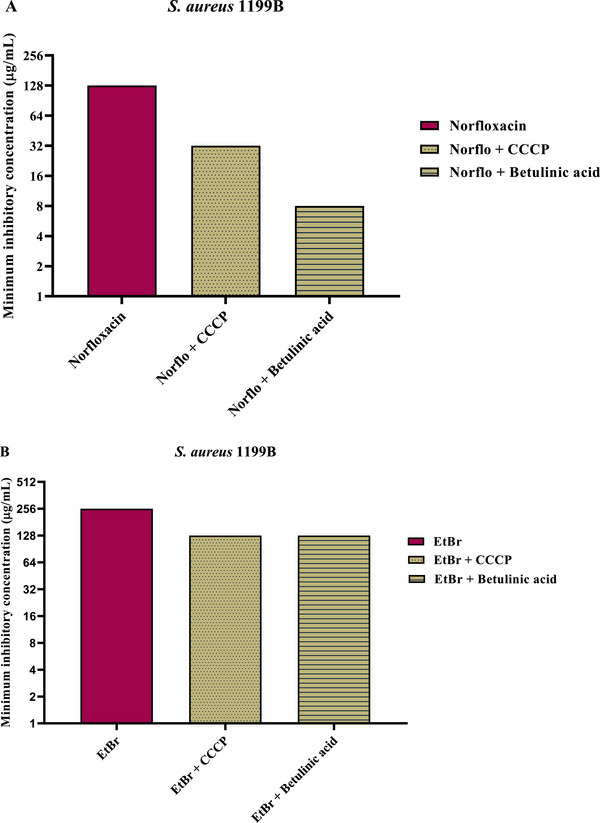
Evaluation of NorA efflux pump inhibition by Betulinic acid against the *S. aureus* 1199B strain. Substance associated with norfloxacin (A) and EtBr (B) in minimum inhibitory concentration (MIC) reduction. Norflo=Norfloxacin, CCCP Carbonyl cyanide m‐chlorophenyl hydrazone, EtBr=Ethidium bromide.

The evaluation of efflux pump inhibition was performed by determining the reduction in the MIC of norfloxacin and EtBr (EtBr), as shown in Figures 1 and 2. For strain SA‐1199 (Figure 1A,B), the combination of BA with norfloxacin promoted a 16‐fold reduction in the MIC (64–4 µg/mL), while for EtBr, the MIC decreased 5‐fold (256–32 µg/mL). The standard efflux pump inhibitor, CCCP, reduced the MIC of norfloxacin 2‐fold (64–32 µg/mL) and that of EtBr 4‐fold (256–64 µg/mL).

For strain SA‐1199B (Figure 2A,B), the MIC of norfloxacin decreased 16‐fold in the presence of BA (128–8 µg/mL), and the MIC of EtBr was reduced 2‐fold (256–128 µg/mL). In comparison, the combination of norfloxacin + CCCP promoted a 4‐fold reduction (128–32 µg/mL), whereas EtBr + CCCP showed a 2‐fold reduction (256–128 µg/mL).

Overall, it is evident that BA displayed superior or equivalent activity to the standard inhibitor CCCP, depending on the strain and substrate analyzed. A compound is considered to have potential efflux pump inhibitory activity when it reduces the MIC of antibiotics that are substrates of these pumps by at least 2‐fold, an assessment that can be complemented by intracellular EtBr accumulation assays (Oliveira‐Tintino et al.).

### 3.3 Evaluation of Efflux Pump Inhibition by Fluorescence Measurement

**FIGURE 3:**
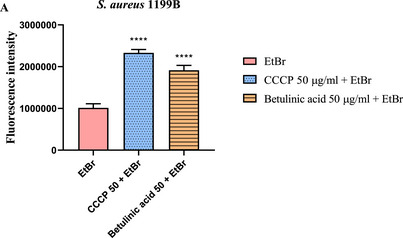
Evaluation of NorA efflux pump inhibition by measuring fluorescence emission in *S. aureus* 1199B strains treated with Betulinic acid in 50 µg/mL. EtBr = ethidium bromide; **** = *p* < 0.0001 versus EtBr.

Fluorescence emission analysis showed that BA at a concentration of 50 µg/mL increased fluorescence by 88.48% compared to the EtBr‐containing control. In addition, CCCP produced an increase greater than 100% (Figure 3).

### 3.4 Evaluation of Bacterial Membrane Permeability Using the SYTOX Green Fluorescence Method

**FIGURE 4:**
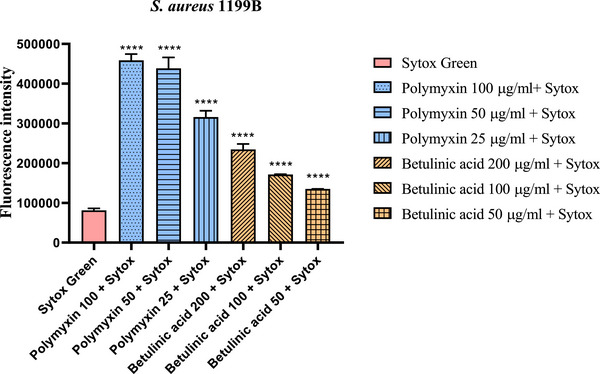
Evaluation of Betulinic acid action on the permeability of the bacterial membrane of *S. aureus* 1199B. ****p < 0.0001 versus Sytox Green. Results are expressed as mean fluorescence intensity. One‐way analysis of variance (ANOVA) followed Tukey's test.

The results of the cytoplasmic membrane permeability assay in SA‐1199B showed that BA at concentrations of 200, 100, and 50 µg/mL increased the fluorescence intensity of SYTOX Green by 188.5%, 110.9%, and 66.0%, respectively, representing a significant increase compared to the negative control (Figure 4).

### 3.5 Molecular Docking

The results obtained showed that the binding energy of ‐9.166 kcal/mol of BA showed a more significant affinity with the homology model of the NorA transporter than that registered with CCCP (‐6.182 kcal/mol), aligning the docking results with the in vitro tests. The energy value calculated for the triterpenoid was also higher than those recorded for norfloxacin (‐7.906 kcal/mol) and EtBr (‐8.965 kcal/mol), the specific and non‐specific substrates of the efflux pump studied, respectively.

**FIGURE 5:**
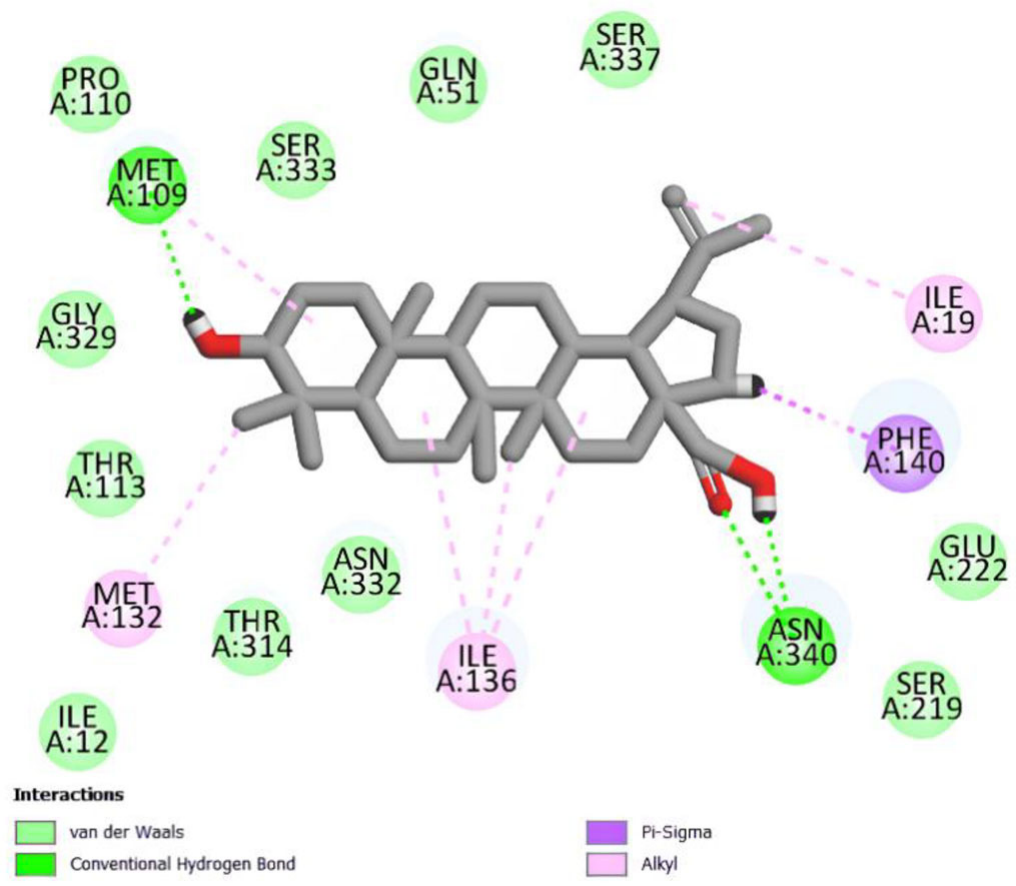
Map of betulinic acid interactions with the NorA model.

The interactions established by BA are classified as hydrogen bonds (Asn340 – 1.87 Å e 2.27 Å; Met109 – 1.86 Å), hydrophobic interactions of the alkyl type (Ile136 – 5.06 Å, 3.84 Å e 5.50 Å; Ile19 – 4.48 Å; Met109 – 4.07 Å; Met132 – 4.30 Å) e type π ‐sigma (Phe140 – 2.92 Å), and van der Waals interactions (around 5.00 Å) (Figure 5).

**FIGURE 6:**
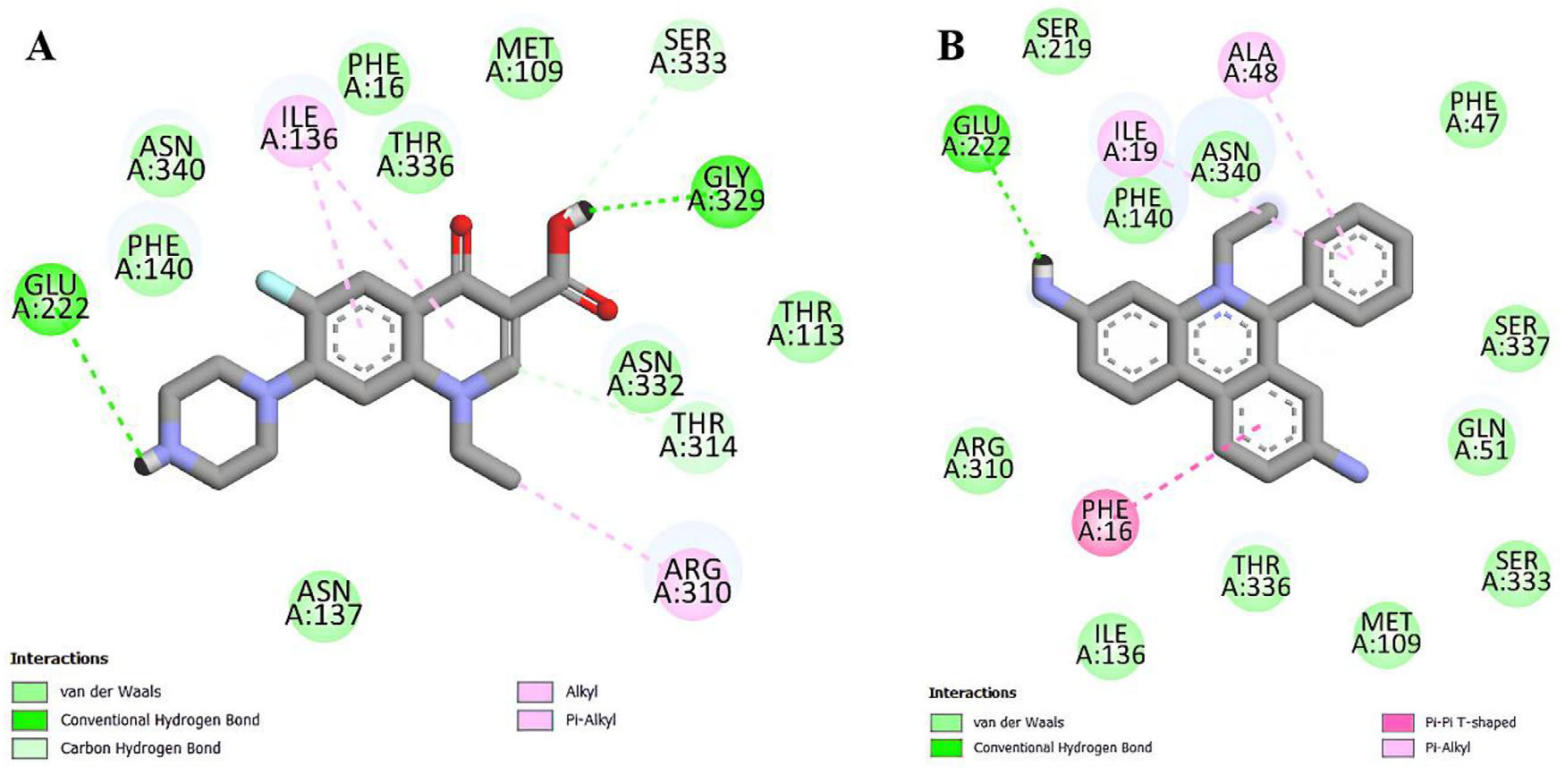
Maps of norfloxacin (A) and ethidium bromide (B) interactions with NorA.

Conventional hydrogen bonds were identified with norfloxacin (Glu222 – 2.95 Å; Gly329 – 2.16 Å), carbon‐hydrogen bonds (Ser333 – 3.06 Å; Thr314 – 3.65 Å), and alkyl‐type hydrophobic interactions (Arg310 – 3.79 Å) and π‐alkyl type (Ile136 – 5.41 Å and 5.15 Å), as well as van der Waals interactions (Figure 6A). Hydrogen bonding (Glu222 – 2.23 Å), hydrophobic interactions of the π‐alkyl type (Ala48 – 4.97 Å; Ile19 – 5.02 Å), and type π—π T‐shaped (Phe16 – 4.90 Å and van der Waals interactions were also carried out by EtBr (Figure 6B).

## 4 Discussion

### 4.1 Antibacterial Activity of BA

This study represents the first investigation into the inhibitory activity of BA on NorA efflux pumps. To date, there have been no reports in the literature exploring this specific function of BA, highlighting the originality and relevance of the results presented here.

Reports on the antibacterial activity of BA vary considerably. Fontanay et al. [29] found no significant antibacterial activity and reported high cytotoxicity associated with the compound. In contrast, Rodrigues et al. [13] extracted BA from *S. joazeiro* and obtained a MIC of 561 µg/mL, an inhibitory concentration but without clinical relevance.

Bilkisu et al. [30] reported a MIC of 0.25 µg/mL for BA extracted from *Psidium guajava*, comparing their results with the standard antibiotic neomycin. Another study demonstrated a MIC value of 12.5 µg/mL against *S. aureus* [31].

### 4.2 Evaluation of Efflux Pump Inhibition by Fluorescence Measurement and Reducing the MIC of Antibiotics and EtBr

Considering the shortage of new antibiotics and the rapid advance of bacterial resistance, research into compounds capable of potentiating conventional antibiotic therapy is a promising strategy for controlling bacterial infections. One example is the clinical use of clavulanic acid in combination with amoxicillin [15].

The most common fluorescent dye used to assess efflux pump inhibitor efficacy is EtBr [32]. The intracellular retention of EtBr enables it to interact with bacterial DNA. As more EtBr remains inside the cell, there is a proportional increase in the bonds with the DNA, which intensifies the fluorescence emitted. When the efflux pumps are blocked, EtBr accumulates internally, causing a significant increase in fluorescence emission [33].

The effects observed in this study are consistent with previous findings from Silva et al. [23], which demonstrated that BA is capable of modulating efflux systems. The compound promoted a significant reduction in the MIC of ciprofloxacin and EtBr in *S. aureus* strains that overexpress the MepA efflux pump.

In a study conducted by [34] with 1199B *S. aureus* strains that overexpress the NorA efflux pump, the pentacyclic triterpene lupeol also demonstrated a similar effect, resulting in an increase in fluorescence emission and indicating greater intracellular accumulation of the marker.

### 4.3 Evaluation of Bacterial Membrane Permeability Using the SYTOX Green Fluorescence Method

SYTOX Green is a fluorescent dye that can be used as an indicator for assessing cell membrane integrity due to its ability to easily penetrate damaged cell membranes, but not permeate the intact cell membrane. It also has advantages such as high sensitivity and binding affinity for nucleic acids within cells [35, 36].

The available literature on the membrane permeability potential of BA is limited, and there are not enough studies to make direct comparisons. Zand et al. [36] investigated the ability of an oleanolic acid (OA) derivative to permeabilize the membrane of methicillin‐resistant *S. aureus*. The results indicated that the OA derivative promoted a significant increase in fluorescence, even at low concentrations, corroborating the findings of this study.

### 4.4 Molecular Docking

Oliveira et al. [37] performed the docking of the triterpenoids α‐amyrin and β‐amyrin with the NorA efflux pump of *S. aureus* and identified the predominance of hydrophobic interactions, including with Phe140, followed by van der Waals interactions and hydrogen bonds. The involvement of hydrophobic NorA residues has also been observed in studies with the terpenes eudesmatriene and totarol, such as methionine, isoleucine, and phenylalanine residues that were included in the results of this study [38, 39].

Transporters of the Major Facilitator Superfamily, such as NorA, have hydrophobic portions as important structural and functional elements, which act, for example, in the recognition of substrates [40]. Therefore, the high affinity in silico and the potentiating effects in vitro may be the result of hydrophobic interactions carried out by BA.

In addition, the docking results with norfloxacin and EtBr showed that their respective binding sites are close to those registered with the triterpenoid evaluated. Thus, the inhibition of the efflux pump by BA may be due to competition for the binding site, one of the main strategies used to block this resistance mechanism [41].

## 5 Conclusion

In conclusion, to our knowledge, this study is the first to evaluate the potential of BA as a NorA efflux pump inhibitor. BA has no intrinsic antibacterial activity against strains SA‐1199 and SA‐1199B. However, BA decreased the MICs of norfloxacin and EtBr, as well as influenced membrane permeability, possibly by inhibiting the NorA efflux mechanism. The increase in EtBr fluorescence emission reinforces the evidence of efflux inhibition. In silico modeling showed that BA has a high affinity for the NorA binding site, suggesting that efflux pump inhibition may result from competition for the binding site. These findings indicate that BA is a strong candidate for clinical application as an efflux pump inhibitor and adjuvant drug in the treatment of bacterial infections associated with efflux mechanisms.

## Author Contributions

**Camila Aparecida Pereira da Silva**: conceptualization. **Nara Juliana Santos Araújo**: research. **Cícera Datiane Morais Oliveira‐Tintino**: methodology. **José Maria Barbosa Filho**: resources. **Gabriel Gonçalves de Alencar**: research. **José Bezerra de Araújo‐Neto**: software. **Josefa Sayonara dos Santos**: research. **Juliete Bezerra Soares**: investigation. **Henrique Douglas Melo Coutinho**: project administration. **Jacqueline Cosmo Andrade‐Pinheiro**: supervision and conceptualization.

## Conflicts of Interest

The authors declare no conflicts of interest.

## Supporting information




**Supporting File 1**: cbdv70799‐sup‐0001‐SuppMat.docx

## Data Availability

Data available on request to the authors.
