# Supplementary material for: Action of Betulinic Acid in the Inhibition of Efflux Pump NorA in Staphylococcus aureus Strains: In Vitro and In Silico Approaches
Source: Chem Biodivers. 2025 Dec 17;23(2):e02869. doi: 10.1002/cbdv.202502869 (PMC12860514; doi:10.1002/cbdv.202502869)
Supplement: Supplementary file 1 — Supporting File 1: cbdv70799‐sup‐0001‐SuppMat.docx [file CBDV-23-e02869-s001.docx]

**Supplementary Material**


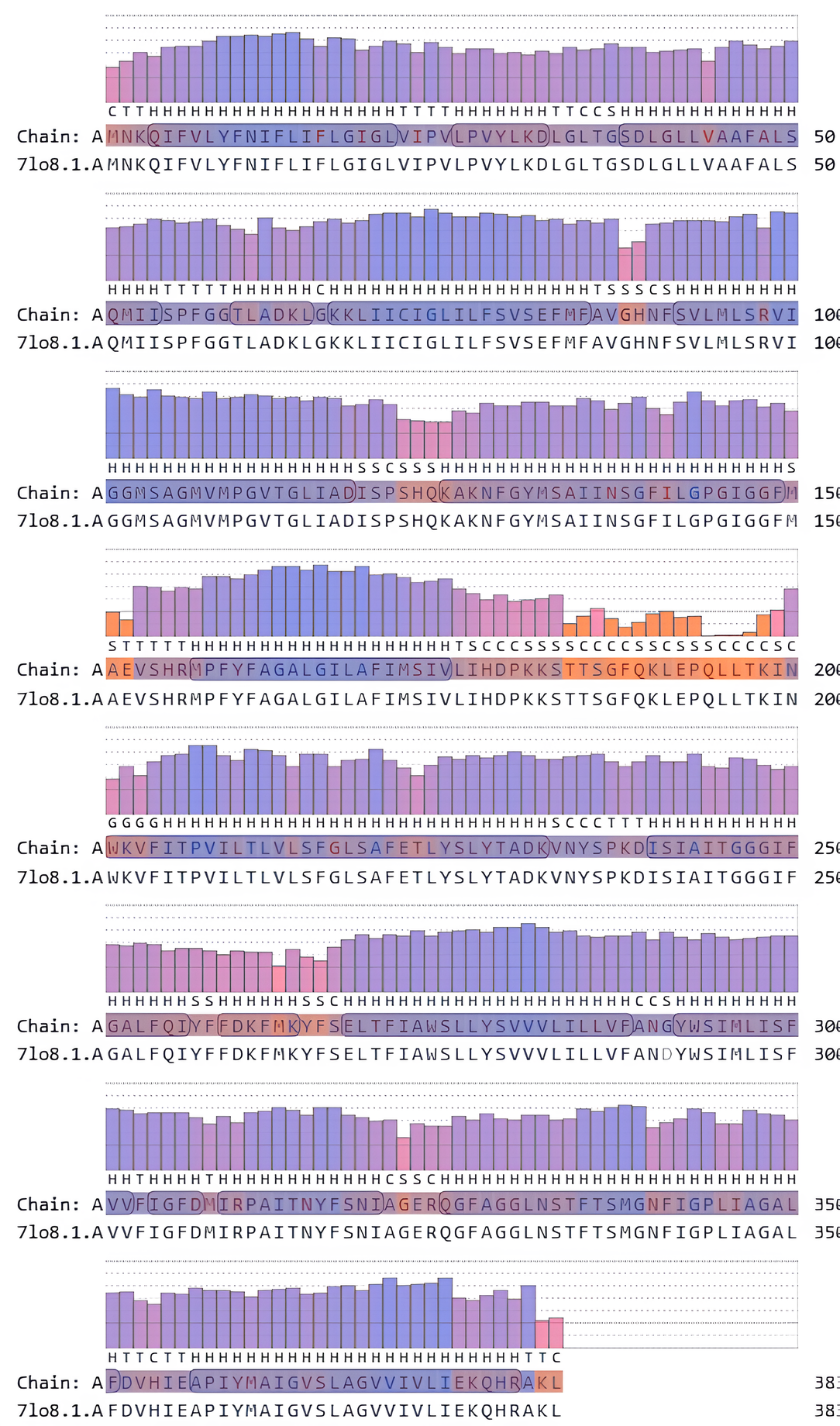


**Figure S1.** Sequence alignment between the NorA homology model and the PDB template: 7LO8


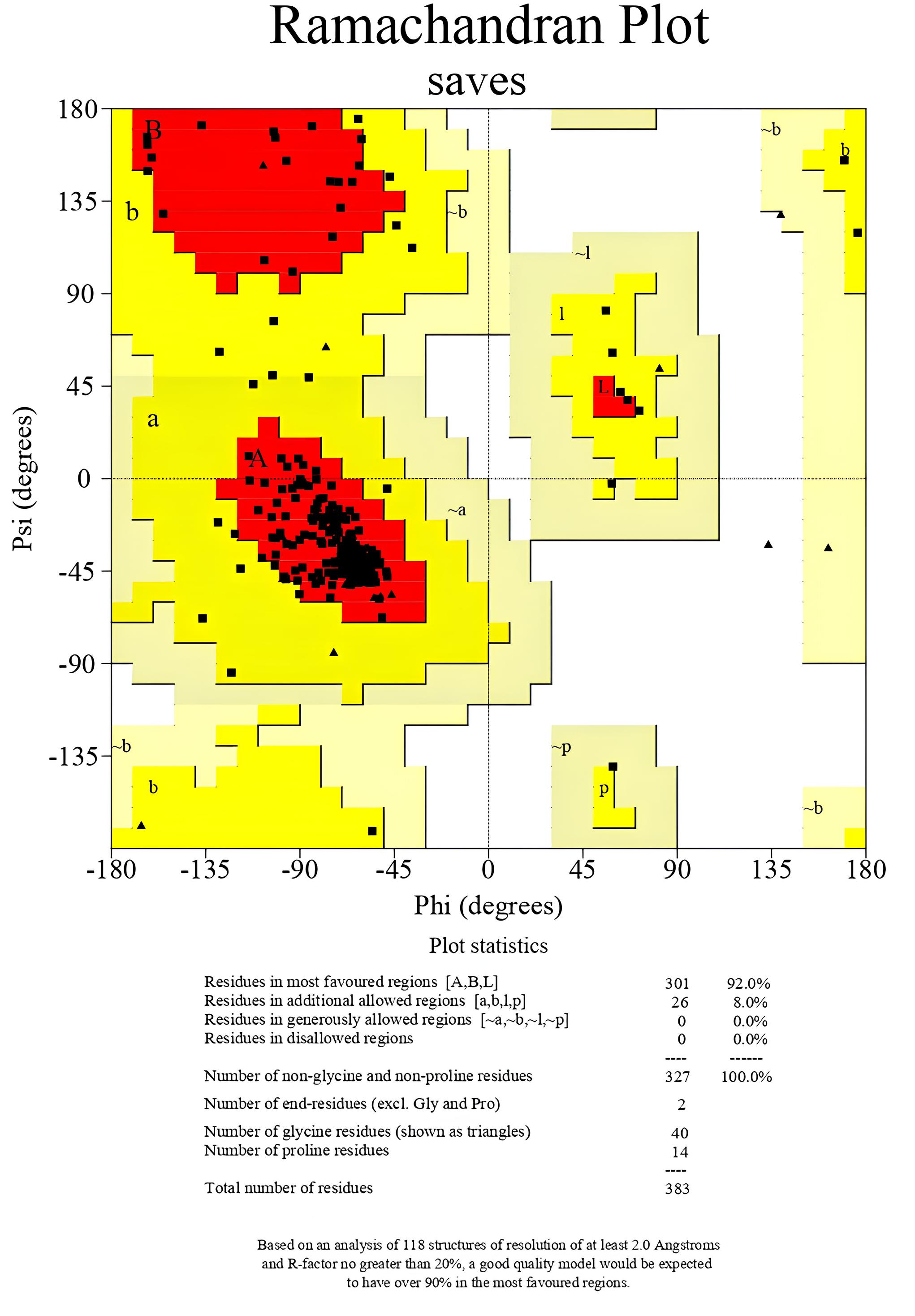


**Figure S2.** Ramachandran plot of the *S. aureus* NorA efflux pump with 100% of residues in allowed regions
